# Stereotactic Body Radiotherapy as an Alternative to Brachytherapy in Gynecologic Cancer

**DOI:** 10.1155/2013/898953

**Published:** 2013-08-13

**Authors:** Gregory J. Kubicek, Jinyu Xue, Qianyi Xu, Sucha O. Asbell, Leslie Hughes, Noel Kramer, Ashraf Youssef, Yan Chen, James Aikens, Howard Saul, Niraj Pahlajani, Tamara LaCouture

**Affiliations:** ^1^Department of Radiation Oncology, Cooper University Hospital, One Cooper Plaza, Camden, NJ 08103, USA; ^2^Department of Gynecology, Cooper University Hospital, One Cooper Plaza, Camden, NJ 08103, USA; ^3^Department of Gynecology, Center for Cancer and Hematologic Disease, Marlton, NJ 08003, USA

## Abstract

*Introduction*. Brachytherapy plays a key role in the treatment of many gynecologic cancers. However, some patients are unable to tolerate brachytherapy for medical or other reasons. For these patients, stereotactic body radiotherapy (SBRT) offers an alternative form of treatment. *Methods*. Retrospective review of patients prospectively collected on SBRT database is conducted. A total of 11 gynecologic patients who could not have brachytherapy received SBRT for treatment of their malignancies. Five patients have been candidates for interstitial brachytherapy, and six have required tandem and ovoid brachytherapy. Median SBRT dose was 25 Gy in five fractions. *Results*. At last followup, eight patients were alive, and three patients had died of progressive disease. One patient had a local recurrence. Median followup for surviving patients was 420 days (median followup for all patients was 120 days). Two patients had acute toxicity (G2 dysuria and G2 GI), and one patient had late toxicity (G3 GI, rectal bleeding requiring cauterization). *Conclusions*. Our data show acceptable toxicity and outcome for gynecologic patients treated with SBRT who were unable to receive a brachytherapy boost. This treatment modality should be further evaluated in a phase II study.

## 1. Introduction

Gynecologic malignancies, mostly consisting of endometrial and cervical cancers, remain common cancers in the United States. For locally advanced cervical cancer, the standard treatment combines chemotherapy (CTX) along with conventional external-beam radiation therapy (EBRT) and a brachytherapy boost (BB) [1–5]. BB is also used in endometrial cancer, in early-stage disease as the sole treatment, and in unresectable and recurrent disease in combination with EBRT [6]. BB is a valuable treatment option because it allows for a high dose to the tumor while sparing the nearby normal structures. To treat to a tumoricidal dose using EBRT alone would lead to significant dose to nearby normal structures (mainly rectum, small bowel, and bladder), which would entail a high likelihood of acute and late toxicity. BB is ideal for treatment of gynecologic cancers because it allows the radioactive source to be placed very close to the target which receives full dose, but because of the inverse-square law (the radiation dose decreases exponentially with distance; so as distance goes from *x* to 2*x*, the radiation dose decreases from *y* to 0.25*y*), the nearby normal tissues receive a much lower dose; thus, BB allows for maximal tumor dose and maximal normal tissue sparing [5, 6]. 

While BB remains the standard treatment option for many gynecologic cancers, there are some patients who are not candidates for this treatment. Patients with comorbid conditions may not be able to tolerate BB, especially an interstitial implant. Also, for patients with unfavorable anatomy, it may not be possible to place BB even with assistance from gynecology oncologists. One possible alternative to BB is stereotactic body radiotherapy (SBRT), which entails high doses of external radiation delivered in a very conformal fashion. While SBRT is commonly used in medically unresectable early-stage lung cancer and has a growing use in other pathologies, there are very little data regarding the role of SBRT used in place of BB. 

Several retrospective clinical reports [7–12] and retrospective dosimetric reports [13] have shown that SBRT appears to be a reasonable treatment option for patients unable to receive a BB. Haas et al. [7] reported on six cervical cancer patients who had anatomic or medical conditions that precluded BB using tandems and ovoids. The patients received an SBRT boost to the cervix instead, using the doses of 20 Gy in five fractions (five patients) and 19.5 Gy in three fractions (one patient). With a median followup of 14 months, there were no reported local failures and no toxicities from the SBRT boost. Molla et al. [8] reported on 16 patients (nine endometrial and seven cervical) who had an SBRT boost to high-risk areas (14 Gy in two fractions for operated patients and 20 Gy in five fractions for nonoperated ones). With a median followup of 12.6 months, there was one patient with a central pelvic recurrence and one patient with late grade-3 rectal toxicity. A retrospective comparative analysis [12] of BB and SBRT plans found that SBRT plans had better target coverage and better dose distributions to normal structures except for bone marrow. 

In order to increase the knowledge pool regarding SBRT as an alternate to BB in certain patients, we are reporting our institution's experience of this treatment modality. 

## 2. Methods

Patients in this study were collected using an IRB-approved prospective radiosurgery database. Clinical information not contained in the database was retrospectively acquired. Eligibility criteria for the review included gynecologic patients who received SBRT but had been referred and had clinical indications for BB. All patients were initially evaluated and had been recommended to undergo a BB, and for medical or other reasons, they were unable to complete the BB and were thus treated with SBRT (Table 1). All patients were evaluated by both gynecology-oncology and radiation oncology; for patients that needed Smit's Sleeve placement, this was performed by the gynecology-oncologist. A total of 11 patients met the eligibility criteria for this review. Median age was 62 years (range: 47–81 years). Seven patients had cervical cancer, two patients had endometrial cancer, and two patients had vaginal cancer. Histology was squamous-cell carcinoma in eight patients, adenocarcinoma in two patients, and carcinosarcoma in one patient. Patients had locally advanced (seven) or recurrent cancers (four patients) but were not metastatic. All patients had conventional external-beam radiation to the pelvis prior to SBRT, three in the form of IMRT and eight in the form of 3D-CRT; dose range for previous EBRT was 45 to 50.4 Gy. Three patients had both EBRT and BB prior to SBRT, two patients had completed EBRT and BB and had local recurrence 1.4 and 1.1 years later, and the other patient had two HDR treatments, but the patient's Smit's Sleeve became misplaced after the second treatment, and when it was not able to be replaced, the patient went on to have SBRT for the remaining treatments. Three patients had surgery (hysterectomy) in addition to previous EBRT; indication for surgery in these patients was for recurrent disease. 

The initial consult note and progress notes were reviewed for the treatment recommendations; in all patients, a BB was recommended. The types of BB that were indicated included tandem and ovoid BB in six patients and interstitial BB in five patients. Reasons for not being able to treat with BB are listed in Table 1. 

Prior to SBRT, all patients had gold fiducial markers implanted for SBRT tracking. It was recommended that four gold-seed fiducial markers be placed, either into the cervix or into the gross tumor if visualized. CT scan for treatment planning was performed without contrast using 1.25 mm slices. Patients were instructed to have a low-residue meal prior to simulation and were coached to have a consistent fluid intake on the day of simulation and the daily treatments to maintain a regular bladder filling rate throughout the treatment. An MRI of the pelvis with contrast was obtained and fused to the planning CT scan for better visualization of the cervix and gross disease; MRI series included T2 weighting with contrast, with and without fat saturation. Prior to SBRT, 7 patients had gross disease present visible on MRI or physical exam.

SBRT target consisted of the gross tumor volume (GTV) in patients with gross disease present prior to SBRT and also a clinical target volume (CTV) for areas thought to be at high risk for residual disease. GTV consisted of gross disease noted on the MRI and physical exam. The CTV would include any extracervical disease at the time of the MRI and physical exam. CTV would also include the entire cervix for patients with cervical cancer with an intact uterus. The CTV was uniformly expanded by 5 mm to create a planning target volume (PTV) to account for a set-up error including rotation. In patients with small intestine or other critical structures adjacent to targets, the PTV was subtracted from the organs at risk to decrease dose to the organs at risk. See Table 2. 

SBRT was delivered in five fractions using CyberKnife (Accuray Incorporated, Sunnyvale, CA, USA) with multiplan planning system version 3.5.4. Numerous noncoplanar beams using 6 MV photons were used for each treatment. Target tracking was performed via the CyberKnife's real-time tracking algorithm; synchrony (respiratory motion software) was not used. CyberKnife tracking, has the ability to track movements (with aid of gold fiducials), and this ability is critical since the cervix is prone to movement [14]. Treatments were delivered every other day, and we feel that normal tissues such as small bowel would tolerate every-other-day better than every-day treatments; every-other-day treatments are also similar to what is used in BB using HDR. Patients were instructed to use Simethicone (Himalaya Drug Company, Bangalore, India) during every day of treatment to reduce bowel distension. None of the patients received chemotherapy concurrent with SBRT, although chemotherapy was typically delivered with EBRT. Treatments were delivered to an isodose line that was aimed for a compromise of target coverage and sparing of the organs at risk. 

Normal tissues were contoured including large bowel, small bowel, femoral heads, sigmoid colon, rectum, bladder, and skin. See Table 2 for more information regarding contouring specifics. Note that the entire organ was contoured as opposed to just the organ wall. For patients that have had previous pelvic radiation to 45 to 50.4 Gy, the point dose limit to the sigmoid colon, rectum, and small bowel was 21 Gy; and the bladder dose limit was 24 Gy. A small volume was allowed to exceed this dose if the normal structures abutted the tumor, in which case 1-2 cc were allowed higher doses up to the target dose. 

After completion of treatment, patients were followed by gynecology oncology as well as radiation oncology, and patients were typically seen 4 weeks after completion of treatment and then every 2 months for the first year; followup visit would include pelvic exam. Imaging (typically MRI) was done at 3 months posttreatment. Toxicity was physician-scored based on CTCAE 4.0. Oncologic outcomes at followup were based on combination of both the physical exam and the followup imaging. 

## 3. Results

### 3.1. Radiotherapy Treatment

Ten patients had five prescribed fractions. One patient who had completed two fractions of HDR BB and then had Smit's Sleeve malposition received three fractions of SBRT. SBRT boost was completed in 10 patients. One patient suffered a stroke after the first fraction and had a subsequent decline in performance status, necessitating the treatment to be discontinued. The stroke was not felt to be related to SBRT. This patient's dosimetric information was not included in the analysis. 

Median SBRT dose for patients completing the treatment was 25 Gy (range: 15–27.5 Gy); the median dose per fraction was 5.0 Gy (range: 4.8–5.5 Gy). Treatments were prescribed to the median 61% isodose line (range: 51%–81%). Median treatment volume was 9,163 cc (range: 1,665–35,740 cc). Median PTV coverage was 88% (range: 71%–94%), and median GTV coverage was 96.5% (range: 90%–97.6%). Median PTV conformality index was 1.5 (range: 1.1–2.9), where conformality index is defined as treated volume divided by PTV. Maximum rectal point dose ranged from 20.8 to 32.6 Gy (median: 23.8 Gy), median rectal dose to 1 cc was 19.6 Gy (range: 18.2 to 27.5 Gy), and median rectal dose to 2 cc was 19.3 Gy (range: 17.6–25.4 Gy). Maximum bladder point dose ranged from 16.5–36 Gy (median: 25.7 Gy), median bladder dose to 1 cc was 20.7 Gy (range: 11.1–22.9 Gy), and median bladder dose to 2 cc was 19 Gy (range: 5.6–16.9 Gy); see Table 3. 

**Figure 1:**
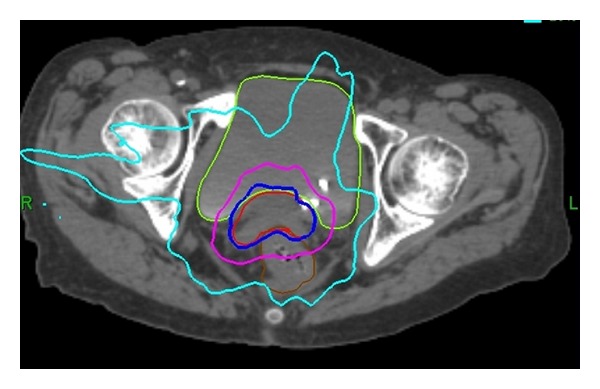
Sample SBRT plan. Typical SBRT plan, patient treated to 25 Gy in five fractions. Bladder, CTV, and rectal contours are shown. Isodose lines include prescription isodose line (75%), pink (50%), and light blue (20%).

**Figure 2:**
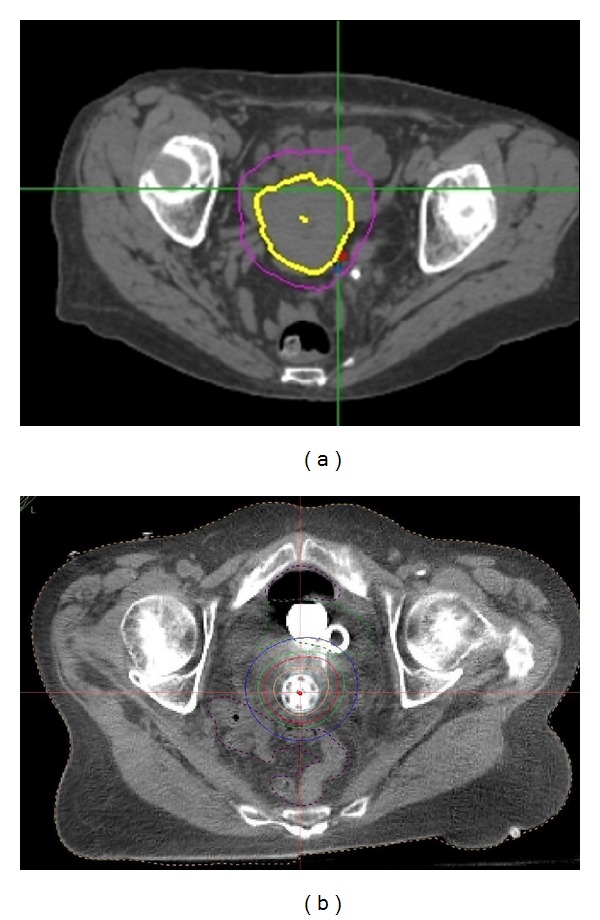
Comparison of SBRT (a) and BB (b). Comparison of target dose using BB and SBRT for the same patient.

A typical SBRT plan is seen in Figure 1. One patient had SBRT after two HDR BB (Smit's Sleeve shifted out of position after the second BB). On comparing her BB and SBRT treatments, maximum normal-tissue point doses were lower for the BB. For this patient, maximum dose per fraction to bladder was 5.8 Gy with SBRT versus 4.45 Gy with BB; for rectum, 5.6 Gy with SBRT versus 5.0 with BB; and for small bowel, 5.3 Gy with SBRT versus 2.8 Gy with BB. Doses to 1 and 2 cc of bladder were 5.2 and 4.9 Gy with SBRT and 5.3 and 4.7 with BB, respectively. Doses to 1 and 2 cc of rectum were 4.9 and 4.5 Gy with SBRT and 4.7 and 4.2 Gy with BB, respectively. Comparison between SBRT and HDR plans is shown in Figure 2. 

The biologically equivalent dose (BED) in terms of equivalent doses given at 2 Gy per day (EQ2) was calculated using the linear quadratic (LQ) equation [15]. The *α*/*β* ratio was taken to be 10 Gy for tumor effects and 3 Gy for late effects. Median EQ2 for the tumor was 31.3 Gy (range: 18.75–35.5 Gy) for the entire group, and median EQ2 for late effects on normal tissues was 40 Gy (range: 24–46.75 Gy), Table 3. 

### 3.2. Oncologic Outcome

At last followup, eight patients were alive. Median followup for all patients was 120 days, and median followup for surviving patients was 14 months. Of the three patients who died, two had recurrent cervical cancer and died of progressive disease; the other had locally advanced cervical cancer and had a stroke during therapy and subsequently went on to hospice care without completing the entire course of treatment. Of the eight surviving patients, one (patient no. 2) had a local recurrence 3.5 years after completion of SBRT (recurrence took place in cervical stump in the region of the previous SBRT). This patient was treated with surgical exoneration and was disease-free after salvage surgery. All of the other patients were disease-free (both local and distant) at last followup. 

### 3.3. Toxicity

During the SBRT boost, two patients were noted to have acute toxicity (grade-2 GU and grade-2 GI, resp.). There was no grade-3 or greater toxicities during the SBRT or within 90 days after SBRT. One patient (patient no. 8) was noted to have late toxicity one year from the completion of SBRT, in the form of mild GI bleeding, and underwent cauterization of the rectal vessels, which resolved the bleeding (GI grade-3 toxicity). The patient who had GI Grade 3 toxicity had a maximum point dose to rectum of 24 Gy, 1 cc dose of 19.4, 2 cc dose of 18.6, and a mean rectal dose of 6.7 Gy, all of which were around the median for all patients treated. There were no other reported late toxicities. 

## 4. Discussion

In the treatment of locally advanced and recurrent gynecologic cancers, chemoradiotherapy and brachytherapy boost (either ring and tandem or interstitial) remain the standard of care. This results in good disease control and acceptable toxicity. Gynecologic Oncology Group (GOG) Trial 120 [1] reported 60% overall survival at 5 years, 22% local progression, and a late toxicity rate (grade 3 or 4) of 1.7% for patients treated with chemoradiotherapy (including intracavity BB) with a median followup of 35 months. With regard to patients treated with interstitial BB, Pinn-Bingham et al. [16] have reported on 116 patients with locally advanced cervical cancer who were not candidates for intracavity BB. Following treatment with external-beam radiation and interstitial BB, they found 85.3% locoregional control and 13% late toxicity rates.

For gynecologic patientss both intracavity and interstitial BB have good outcomes and are important components of the standard treatment for these patients [1–6, 16, 17]. However, some patients are unable to receive BB, because of either unfavorable anatomy or comorbid conditions. For these patients, treatment options become more limited. Conventional EBRT has been tried in place of BB with overall poor results. Barraclough et al. [18] have reported on 44 patients treated with external-beam boost instead of BB (“technical limitations” was listed as the reason for not providing BB in 73% of patients) and found a 48% recurrence rate with a median followup of 2.3 years. While this treatment is likely better than no boost at all, the importance of a high recurrence rate of the disease is secondary to dose limitations of normal pelvic tissue with conventional EBRT.

Another possible option for patients who are not BB candidates is SBRT. SBRT has advantages over conventional radiotherapy in being able to deliver higher doses while minimizing normal tissue radiation exposure. This allows the tumor to receive a higher biologically equivalent dose than with conventional radiation. Our results for SBRT showed good local control and a medically acceptable toxicity. Among the surviving patients (nine patients), only one had a local recurrence. Two patients with recurrent disease had persistent disease after SBRT, and they continued to have disease progression despite SBRT, and they subsequently died from the disease. Our toxicity results were also determined as tolerable with only one grade-3 toxicity (grade-3 GI) reported. 

The data on this topic are limited to a few retrospective series, and our results mirror the available literature on SBRT in place of BB. Haas et al. [7] have reported on six cervical cancer patients treated with SBRT boost (median of 20 Gy in five fractions) after CRT. Their series did not find any local recurrences and did not report any toxicity with a median followup of 14 months. Molla et al. [8] have reported on 16 patients (nine endometrial and seven cervical cancers, while 15 patients had a hysterectomy) treated with SBRT (14 Gy in two fractions for postoperative and 20 Gy in five fractions for nonoperated patients) instead of BB. This report found one patient with late rectal bleeding (GI grade-3 toxicity) and 1 recurrence with a median followup of 12.6 months. Guckenberger et al. [12] have reported on 19 patients with recurrent cervical or endometrial cancer, who had pelvic sidewall involvement or large tumors not amenable to BB. SBRT consisted of 15 Gy in three fractions, with a median followup of 22 months. Three-year overall survival was 34%, and local control was 81%; two patients had grade-4 intestinovaginal fistulae, and one had a grade-4 small bowel ileus. Kemmerer et al. [10] reported on 11 patients with stage unresectable I-III endometrial cancer treated with SBRT boost of 30 Gy in 5 fractions; they found no late toxicity and 55% local-regional control. Higginson et al. [11] reported on a heterogeneous group of 5 with a range of SBRT doses, one of whom had late grade-3 rectal bleeding after receiving 20 Gy in 5 fractions. 

All of the data to date are retrospective and heterogeneous but some trends do emerge. First of all, there appears to be good local control with this treatment modality. Secondly, the major late toxicity seen in our series and that of several others [8, 11] have been late GI toxicity, while the data thus far do not allow a precise calculation of dose tolerance for the rectum; it is important that this toxicity be discussed with patients and that there be attempts to constrain dose to the rectum. 

At this point, it is not standard of practice to replace BB with SBRT in patients who are BB candidates. However, we have shown that, when BB is not an option, SBRT can be a safe and effective treatment modality. Further work in this area can be used to better define SBRT dose and to prospectively collect toxicity and outcome information on this patient subset. Our current treatment protocol is to treat to 25 Gy in five fractions using dose constraints as described previously. 

## 5. Conclusions 

Brachytherapy implants remain the standard of care as a method to deliver radiation boost for gynecological cancers. However, for patients unable to have a brachytherapy procedure, we found acceptable toxicity and outcome with SBRT. This treatment modality should be further evaluated in a phase II study, with dose and fractionation of 25 Gy in 5 fractions, and close adherence to organ at risk limits.

## Figures and Tables

**Table 1:** Patient characteristics.

Patient #	Diagnosis	Stage	Histology	Recommended implant	Reason that implant could not be performed
1	Cervical	Recurrent	Scc	Interstitial	Size and location
2	Cervical	Recurrent	Carcinosarcoma	Interstitial	Bleeding
3	Endometrial	Recurrent	Adenocarcinoma	Interstitial	Difficulty with interstitial, short endocervix makes tandem difficult
4	Vaginal	T2	Scc	Interstitial	Proximity to bladder and bowel
5	Vaginal	T2	Scc	Interstitial	Difficulty w visualization of tumor
6	Cervical	IIIb	Scc	Tandem and ovoid	Comorbid conditions
7	Cervical	IIIb	Scc	Tandem and ovoid	Unable to place Smits Sleeve
8	Cervical	IIIb	Scc	Tandem and ovoid	Unable to place sleeve
9	Cervical	IIIb	Scc	Tandem and ovoid	Smits Sleeve became misplaced
10	Cervical	Recurrent	Scc	Tandem and ovoid	Unable to place sleeve
11	Endometrial	T2N1	Adenocarcinoma	Tandem and ovoid	Smits Sleeve perforation through uterus

Scc: squamous cell carcinoma.

**Table 2:** Target and OAR definitions.

Structure	Definition
GTV	Gross tumor as visualized on MRI (T2) and physical exam
CTV	Entire cervix for most patients, including extracervical disease if present on pre-SBRT MRI
PTV	CTV plus expansion of 5 mm (using less of an expansion if adjacent to organs at risk)
Rectum	Entire rectum, superior limit will be rectosigmoid junction
Bladder	Entire bladder and contents
Small bowel	Bowel loops up to 2 cm above target

**Table 3:** Treatment characteristics.

Patient	Previous RT (EBRT and HDR)	CTV volume (mm^3^)	SBRT dose/Fx	SBRT dose equivalent (2 Gy/fx) alpha/beta 10	SBRT dose equivalent (2 Gy/fx) alpha beta 3
1	45 + 30 Gy HDR	254485.7	27.5/5	35.52083	46.75
2	45	9893.03	25/5	31.25	40
3	50.4	34818.04	25/5	31.25	40
4	45	19947.13	25/5	31.25	40
5	45	40721.84	22.5/5	27.1875	33.75
6	45	129132.1	5/1	6.25	8
7	45	174427.2	25/5	31.25	40
8	45	11549.91	24/5	29.6	37.44
9	45 + 12 HDR	16655.7	15/3	18.75	24
10	45+ 30 HDR	143710.1	25/5	31.25	40
11	45	45809.38	25/5	31.25	40
